# Crowding-induced Cooperativity in DNA Surface Hybridization

**DOI:** 10.1038/srep09217

**Published:** 2015-04-15

**Authors:** Qun-li Lei, Chun-lai Ren, Xiao-hang Su, Yu-qiang Ma

**Affiliations:** 1 National Laboratory of Solid State Microstructures and Department of Physics, Collaborative Innovation Center of Advanced Microstructures, Nanjing University, Nanjing 210093, China; 2 Center for Soft Condensed Matter Physics and Interdisciplinary Research, Soochow University, Suzhou 215006, China

## Abstract

High density DNA brush is not only used to model cellular crowding, but also has a wide application in DNA-functionalized materials. Experiments have shown complicated cooperative hybridization/melting phenomena in these systems, raising the question that how molecular crowding influences DNA hybridization. In this work, a theoretical modeling including all possible inter and intramolecular interactions, as well as molecular details for different species, is proposed. We find that molecular crowding can lead to two distinct cooperative behaviours: negatively cooperative hybridization marked by a broader transition width, and positively cooperative hybridization with a sharper transition, well reconciling the experimental findings. Moreover, a phase transition as a result of positive cooperativity is also found. Our study provides new insights in crowding and compartmentation in cell, and has the potential value in controlling surface morphologies of DNA functionalized nano-particles.

DNA hybridization/dehybridization (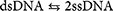, ds: double-stranded, ss: single-stranded) is an important biological process for genetic functions of cell. In recent years, there are increasing interests in using dense DNA brush to model cellular crowding^1^ and compartmentation^2^,^3^,^4^. DNA functionalized surfaces have also attracted intensive attentions in areas of bimolecular detection, gene therapy and nano-material. For example, DNA microarrays^5^ are widely used for DNA sequencing; DNA functionalized nanoparticles, or Spherical Nucleic Acids (SNA)^6^, are designed as vectors for gene delivery^7^,^8^ or as basic units for programmable colloid self-assembly^9^,^10^.

The DNA brush system can create genetic molecular density approximating cellular value (10^7^ bp/*μm*^3^) or higher^4^. Such a crowded environment leads to distinctive cooperative behaviors in DNA hybridization. Experiments^11^,^12^,^13^ showed that the width of hybridization/melting transition for planar DNA brush is broader than that in solution. This implies that preceding hybridized DNA impede succeeding hybridizing process, a typical negatively cooperative behaviour^14^. However, a ‘sharp melting’ transition was also observed in surface-surface hybridization between DNA-coated nanoparticles, raising the discussion of existence of positively cooperative hybridization, where individual hybridizing events are mutually facilitated^15^. This problem is further complicated in a microcantilevers experiment, where Wu et al.^16^ found that under certain conditions surface tension goes down during the process of DNA hybridization.

Another issue deeply related to the hybridization cooperativity is the possible phase separation in dense DNA brush. Since dsDNA and ssDNA are very different in length, size, charge and conformation, it is interesting to ask whether hybridization can cause phase separation in such a crowded condition. Similar phase behaviors have been observed in other systems^17^,^18^. Researches in this area not only can provide new ideas in explanation of the emergence of compartments in cell^1^, but also enables us to design surface morphologies through specific DNA-DNA hybridization. Recently, DNA patchy particles^19^,^20^,^21^ have shown such potentials.

There have already been several models which focus on either negative^22^,^23^,^24^ or positive^15^,^25^,^26^ cooperativity under different scales and based on distinct mechanisms. However, whether both positive and negative cooperativity can be explained within the same framework at the molecular level and how the molecular crowding influences the cooperativity are currently unaddressed. In our theory, molecular crowding is well described by explicitly taking into account the size, shape, charge and conformation of different species, and various subtle interactions among them are included. We find that molecular crowding can lead to both positively and negatively cooperative hybridization by completely different mechanisms. A first-order phase separation is also found as a result of positively cooperative hybridization, and the dsDNA-rich and ssDNA-rich coexisting phases are obtained.

## Results

### Quantification of cooperativity and two distinctive cooperative behaviours

**Figure 1:**
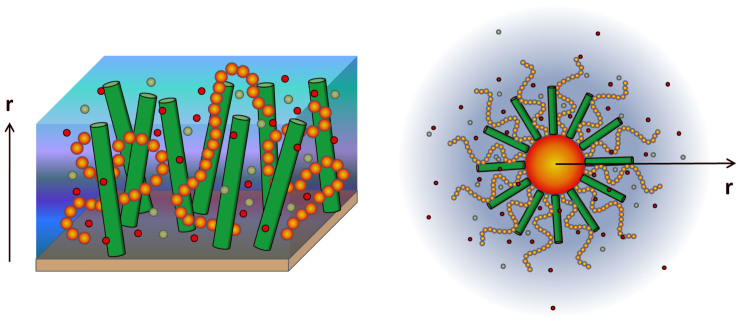
Schematic of two DNA surface systems: planar surface (left) and spherical surface (right). Anchored green rods represent dsDNA while yellow sphere-linked chains represent ssDNA. Red and green small spheres are cations and anions respectively.

Cooperative hybridization arises because different grafted DNA molecules begin to interact with each others. If DNA molecular density is low, hybridization is non-cooperative and the hybridization curve obeys the classical Langmuir isotherm^22^,^27^ 
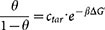
, with the hybridization fraction *θ*, the target ssDNA concentration *c_tar_* and the surface hybridization free energy Δ*G*′. This isotherm fails when the interactions between grafted DNA emerge (See Figure 1). In order to study the cooperative hybridization in crowding condition, a general expression of DNA hybridization isotherm that incorporates complex intermolecular interactions is needed. This task is accomplished by using the molecular theory (see Supplementary section 1 and 2). The result is rather simple:
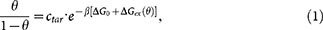
with the standard hybridization free energy in DNA solution Δ*G*_0_. Equation (1) differs from classical Langmuir isotherm mainly in the excess hybridization free energy Δ*G_ex_*(*θ*), which satisfied Δ*G_ex_* = *U_ds_* − *U_ss_*, with *U_ds_* and *U_ss_* the potentials of mean force (PMF) of dsDNA and ssDNA molecules staying in DNA layer, respectively. The PMF reflects the crowdedness of the layer, which includes isotropic excluded volume and orientational interactions between DNA molecules, ionic osmotic pressure, electrostatic interaction, hydration repulsion among dsDNA and entropic force arising from DNA conformational deformation (see Supplementary section 3).

**Figure 2:**
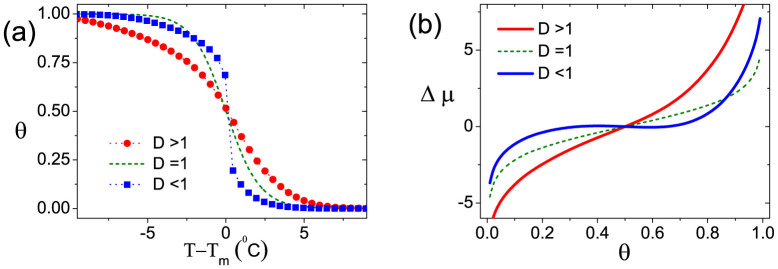
Quantification of cooperative DNA hybridization. (a) Melting curve of DNA hybridization. (b) Exchange chemical potential Δ*μ* of DNA hybridization. Non-cooperativity situation (*D* = 1) is calculated by using classical Langmuir model. Positive (*D* < 1) and negative (*D* > 1) cooperativity situations correspond respectively to ***A*** and ***C*** points in phase diagram Figure 3. Here three melting curves are shifted together for the convenience of comparison. The melting temperatures *T_m_* are: 50.5*°C* for *D* > 1; 69.5*°C* for *D* = 1; 66.4*°C* for *D* < 1. The 30 mer DNA probe sequence used to calculate the melting curve is 5′-TTG TAA ATT CTG CAA GTG ATA ATA TAG AAA-3′ (Δ*H*_0_ = −222.1 *kcal mol*^−1^, Δ*S*_0_ = −619.6 *cal mol*^−1^ · *K*^−1^, *c_tar_* = 100 *nM*).

The purpose of this work is to investigate cooperativity of DNA hybridization in dense DNA layer. A common measurement of hybridization cooperativity is the full width at half-maximum (FWHM) of its hybridization or melting curve. For positive cooperativity, the transition will be sharper, leading to a narrower FWHM. Oppositely, negative cooperativity retards the transition, resulting in a broader FWHM, as demonstrated in Figure 2(a). The FWHM can be defined as 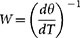 at the melting point *T_m_* (*θ* = 0.5). For DNA brush system, it can be written as
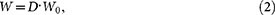
where *W*_0_ is a reference value representing the melting width in DNA solution^28^ (no intermolecular interaction). Hence, *D* is a normalized melting width. *D* = 1, *D* < 1 and *D* > 1 can be used to represent non-cooperativity, positive cooperativity and negative cooperativity respectively. According to equation (1), *D* can be expressed as
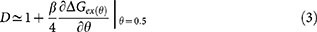


The interpretation of equation (3) is simple. For non-cooperative situation, DNA hybridizations or dehybridizations happen independently, hence Δ*G_ex_* is a constant and *D* = 1. Cooperativity happens when Δ*G_ex_* varies with *θ*. More specifically, if increasing *θ* lowers the excess hybridization free energy, it is positive cooperativity, meaning that hybridization or dehybridization events in DNA layer are mutual-facilitated. In this situation we can easily get *D* < 1. Inversely, if Δ*G_ex_*(*θ*) is an increasing function of *θ*, it is negative cooperativity, indicating that hybridization or dissociation events are mutually impeded. In this case, we will have *D* > 1.

In fact, *D* can be also written into a more general form
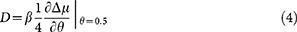
where Δ*μ* is the exchange chemical potential^23^ of single DNA molecule from coil to helix state Δ*μ* = *μ_ds_* − *μ_ss_*. Equation (4) facilitates us to measure the cooperativity by Δ*μ* instead of plotting the melting curve. The comparison between Δ*μ* − *θ* curve and melting curve is given in Figure 2(b). Moreover, a negative gradient of Δ*μ* is an indicator of phase transition. Therefore, equation (4) also enables us to identify phase separation by testing whether *D* < 0. Equation (3) and (4) also imply that hybridization cooperativity is insensitive to the specific DNA sequences and other factors which are unchanged during hybridization process. The insensitivity to sequence is verified by melting curves for four more DNA sequences with different G-C contents as shown in Supplementary Figure S1.

### Phase diagram of cooperativity

**Figure 3:**
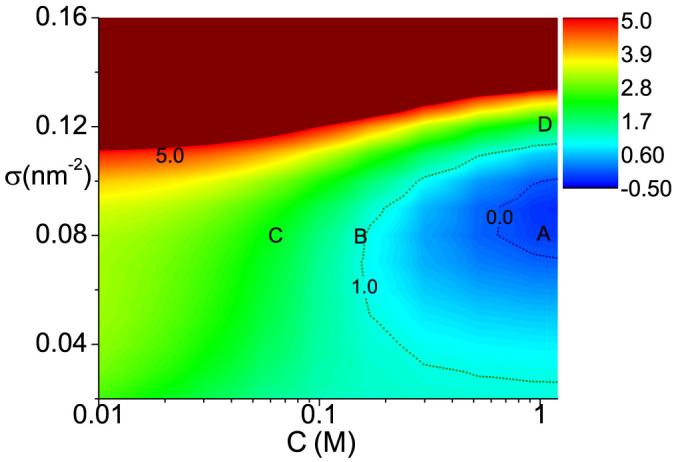
Cooperativity phase diagram of DNA hybridization for planar surface. Cooperativity is characterized by the normalized melting width *D*. DNA length *N* = 30. Positive cooperativity area and phase separation area are enclosed by dot lines of *D* = 1 and *D* = 0 respectively. ***A***, ***B*** and ***C*** points correspond to three ion concentrations: *C* = 1 *M*, *C* = 0.15 *M* and *C* = 0.05 *M* with the same molecular density of *σ* = 0.08 *nm*^−2^. ***D*** points corresponds to molecular density *σ* = 0.12 *nm*^−2^ at *C* = 1 *M*.

In Figure 3, we show the phase diagram as functions of ion concentration and DNA molecular density, where cooperativity is characterized by normalized melting width *D*. DNA length is fixed as N = 30. Regions of positive cooperativity and negative cooperativity are separated by the dot line of *D* = 1, and phase transition happens in the area of *D* < 0. We can find that negative cooperativity dominates in the cases of either low ion concentration or very high molecular density, while positive cooperative hybridization mainly occurs in the region of high ion concentration and middle molecular density. Moreover, within the positive cooperativity region, phase transition occurs in the area of *D* < 0.

### Effect of ionic strength

**Figure 4:**
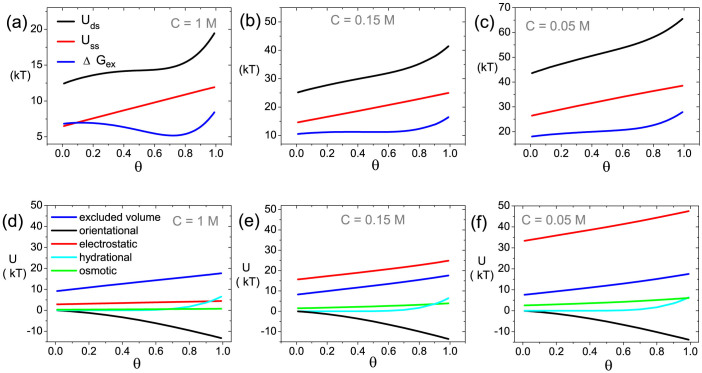
Energetic analysis of cooperative hybridization under different ion strengths. Excess hybridization energy Δ*G_ex_*(*θ*) and PMF of dsDNA (*U_ds_*) and ssDNA (*U_ss_*) as functions of hybridization fraction *θ* for (a) *C* = 1 *M*, (b) *C* = 0.15 *M* and (c) *C* = 0.05 *M*. Contributions to dsDNA's PMF from various interactions for (d) *C* = 1 *M*, (e) *C* = 0.15 *M* and (f) *C* = 0.05 *M*. The legend for (d)–(f), from the top down, indicates isotropic excluded volume interaction, orientational interaction, electrostatic interaction, hydration repulsion and ionic osmotic pressure.

In our model, we assume the DNA layer is immersed in the solution with the salt of NaCl. To study the effect of ion strength, from high to low, we choose three different ion concentrations of *C* = 1, 0.15, 0.05 *M*, corresponding to ***A***, ***B***, ***C*** points marked in phase diagram Figure 3. All three points share the same molecular density of *σ* = 0.08 *nm*^−2^. Melting curves of ***A*** and ***C*** points have already been plotted in Figure 2(a), showing distinct positive and negative cooperativity. This can be further tested by the trends(decreasing or increasing) of corresponding Δ*G_ex_*(*θ*) near *θ* = 0.5, as shown in Figure 4(a), (c). As for ***B*** point, Figure 4 (b) shows an almost flat curve of Δ*G_ex_*(*θ*) at *θ* = 0.5, which implies very weak cooperativity in this case. It agrees the results shown in Figure 3, where B is very close to the *D* = 1 curve.

PMF of dsDNA and ssDNA are also given in Figure 4(a)–(c), since Δ*G_ex_* = *U_ds_* − *U_ss_*. We can expect that when *θ* increase, the layer becomes more and more crowded. So both *U_ds_* and *U_ss_* go up along with *θ*. However, the increase of *U_ds_* at high ionic strength(1 *M*) obviously slows down with a modest *θ*, indicating that some part of repulsions felt by dsDNA are relaxed during the hybridization process. This is the reason for the decrease of corresponding Δ*G_ex_*(*θ*). To find the causation of this crowding relaxation, it is necessary to evaluate different contributions to the crowdedness separately.

In our model, molecular crowding is explicitly depicted by isotropic excluded volume and orientational interactions between DNA molecules, electrostatic interaction, ionic osmotic pressure and hydration repulsion among dsDNA in the layer. In particular, the orientational interaction between dsDNA can be viewed as attractive since it can reduce the free energy by aligning dsDNA in the same direction. The advantage of our model in such complex system, is that we can decompose the molecular crowdedness based on different mechanisms. Figure 4(d)–(f) show contributions to PMF of dsDNA molecule from different interactions under different ionic strengths. At high ion concentration(1 *M*), electrostatic interaction is rather weak due to the strong electrostatic screening. Therefore, isotropic excluded volume repulsion and anisotropic orientational attractions are the most two important interactions to determine the collective behavior of DNA molecules. Both of them increase with the formation of more dsDNA. However, orientational attraction increases faster, which leads to the decrease of Δ*G_ex_*(*θ*) and the positive cooperativity. As the ion strength decreasing, electrostatic repulsions get stronger (Fig. 4(e)) and finally play the dominating role (Fig 4(f)), resulting in negative cooperativity. Therefore, by varying the ion strength from high to low, we show how the electrostatic interaction competes with orientational attraction, and leads to two completely different cooperative hybridization behaviours. It should also be mentioned that, for all three cases, when *θ* is close to 1, hydration repulsions rise quickly, making a faster increase of Δ*G_ex_*(*θ*) near *θ* = 1.

### Effect of DNA molecular density

**Figure 5:**
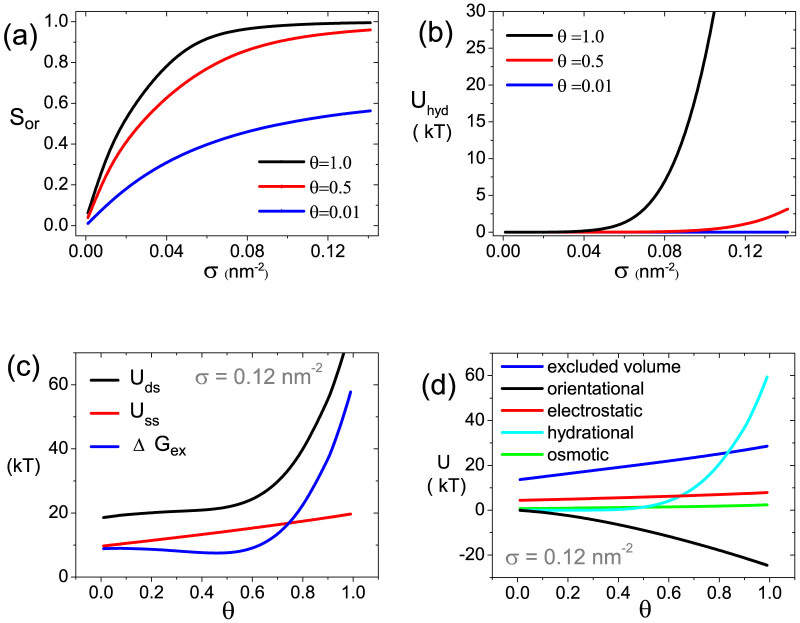
Effects of DNA molecular density. (a) Orientational order parameter *S_or_* and (b) contribution of hydration repulsion to dsDNA's PMF, as functions of molecular density under different *θ* = 0.01, 0.5, 1.0, for high ion concentration (*C* = 1 *M*). (c), (d): Energetic analysis for ***D*** point in phase diagram Figure 3 (*σ* = 0.12 *nm*^−2^, *C* = 1 *M*).

DNA molecular density, or surface coverage, which directly relates to molecular crowding, is another crucial factor to determine the cooperative hybridization. As disclosed by the phase diagrams, increasing the molecular density(crowdedness) has a non-monotonic effect on the hybridization cooperativity under high ion strength condition. From low molecular density (*σ* ≤ 0.02 *nm*^−2^) to moderate molecular density (*σ* ~ 0.08 *nm*^−2^), the increasing crowdedness significantly enhances the orientational attraction as reflected by a fast increasing of orientational order parameter 
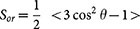
 given by Figure 5(a). This makes the hybridization cooperativity change from non-cooperativity to positive cooperativity. However, further increasing the molecular density from moderate molecular density to high molecular density (*σ* > 0.1 *nm*^−2^) shifts the hybridization cooperativity from positive to negative. This is because under such highly crowded condition, DNA molecules get much closer to each other, short range hydration repulsion among dsDNAs begins to play the dominant role as shown in the Figure 5(b). Such a dramatic increment of hydration repulsion with *θ* overwhelms that from orientational attraction, resulting in negative cooperativity. The threshold *σ* = 0.08 *nm*^2^ for the strong hydration repulsion agrees with experimental observation of Levicky's group^29^,^30^. This conclusion is verified by the energetic analysis for high molecular density *σ* = 0.12 *nm*^−2^ at ion concentration of 1 *M* (***D*** point in phase diagram Fig. 3) in Figure 5(c),(d). Compared with the energetic analysis of ***A*** point, a significantly enhanced hydration repulsion caused by increasing molecular density wins the orientational attraction and makes 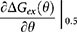 change from negative to positive, which results in the negative cooperativity.

### First order phase transition

From above analysis, we can conclude that both negatively and positively cooperative hybridization arise from molecular crowding. Negative cooperativity (*D* > 1) happens when crowding-strengthened repulsive interactions play the dominate roles, while positive cooperativity (*D* < 1) occurs when the entropy-favouring orientational attraction becomes more important. Moreover, a phase transition can happen in the region of positive cooperativity (*D* < 0). This kind of phase transition is a coupling between the helix-coil transition and nematic-isotropic transition^31^, which has been verified in polypeptide solutions^32^,^33^. Recently, short dsDNA molecules were also found to form domains in high density ssDNA solution^17^.

**Figure 6:**
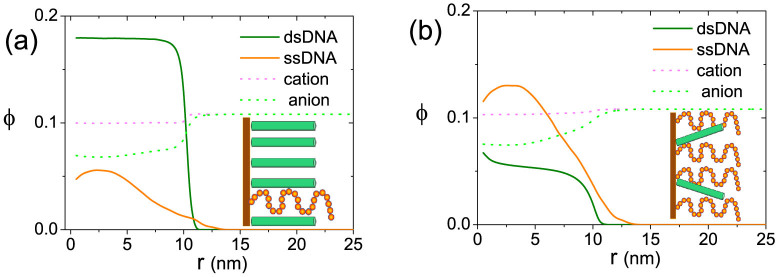
DNA and ion distributions of two coexisting phases. (a) dsDNA-rich state; (b) ssDNA-rich state. *C* = 1 *M*, *σ* = 0.08 *nm*^−2^, *N* = 30.

Generally speaking, there is no spontaneous isotropic-nematic phase transition in hard rod brush systems^34^,^35^. Here we show that the phase transition occurring in the DNA layer is a first-order one which can lead to a two-phases separation. In Figure 6, detailed structural informations are given for the two coexisting phases: dsDNA-rich state (a) and ssDNA-rich state (b). An interesting phenomenon is that ions are repelled from the DNA layer due to strong molecular crowding. For the charge neutrality of the system, anions are repelled much more than cations. Moreover, it's well known that for simple solid brush systems, only microscopic phase separation is possible due to the immobility of anchored chains^36^. In our system, however, the positions of dsDNA are mobile despite the immobility of anchored ssDNA as a result of the dynamic equilibrium of DNA hybridization/dehybridization. Calculated surface tensions near the transition point also show positive value(see Discussion Section). These suggest that the phase separation that can happen in our system is a macroscopic one, completely different from that of classical solid brush systems.

## Discussion

In practical applications, surface curvature and chain length are two controllable factors. Experimental evidences have shown that accommodation of DNA on spherical nano-particles can be quite different from that of planar surface^37^. In view of the enormous applications of spherical nucleic acids, it is an important issue to understand how surface curvature influences molecular crowding and DNA cooperative hybridization.

**Figure 7:**
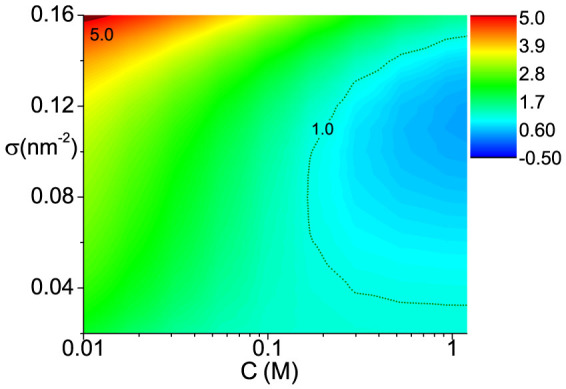
Cooperativity phase diagram for the spherical surface. The radius of sphere is 10 nm. DNA length *N* = 30. Others are the same as phase diagram Figure 3.

In Figure 7, we show the cooperativity phase diagram of DNA hybridization on a spherical surface with radius of 10 nm. Compared with planar surface, intensity of cooperativity is remarkably reduced for both positive and negative cooperativity regions. This indicates that the molecular crowding is weakened since spherical surface has a larger spatial accessibility for DNA. As a result, phase separation is suppressed. However, the region of positive cooperativity is expanded to the area with high molecular density. Therefore, on the whole, high curvature favors positive cooperativity. In addition, high curved sphere has much smaller surface area compared with planar surface, which can effectively resist the surface heterogeneity^12^ and avoid the kinetic trap^38^. All these factors could attribute to a smaller change of width of melting curve and a stronger binding affinity of DNA on nano-particle surface^7^,^39^.

**Figure 8:**
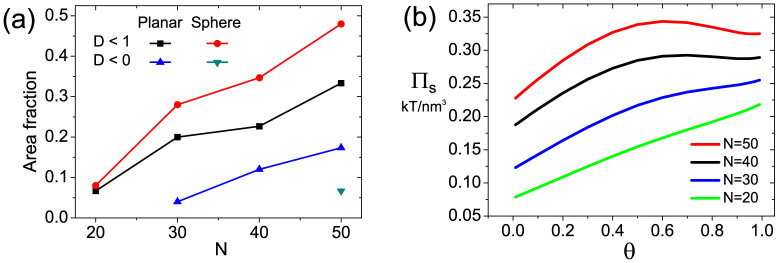
Effects of DNA chain length. (a) Area fractions of positive cooperativity and phase transition areas in phase diagrams for different DNA lengths (Supplymentray Fig. S2). (b) Surface tension Π*_s_* as a function of *θ* under the condition of *σ* = 0.07 *nm*^−2^ and ion concentration *C* = 1 *M*.

To investigate how DNA length affects cooperativity, we also calculate the cooperativity phase diagrams for N = 20, 40, 50 (see Supplementary Fig. S2) for both planar and spherical surfaces. We give the statistics on area fractions of positive cooperativity (*D* < 1) and phase transition (*D* < 0) in Figure 8(a). It can be found that increasing chain length can significantly enlarge the region of positive cooperativity and phase transition owing to enhanced orientational attraction. In Figure 8(b), we also plot surface tension as a function of *θ* for different DNA lengths under the condition of *σ* = 0.07 *nm*^−2^ and ion strength of 1 *M* for the planar surface. We find that the surface tension increases monotonously in short length case. For the long DNA case, there is a maximum value followed by a drop when *θ* approaching 1. This decrease of surface tension is a result of crowding relaxation due to a more ordered dsDNA alignment. Our finding is qualitatively consistent with experimental observation^16^ and other theoretical calculations^40^,^41^.

In reality, many other factors may influence DNA hybridizing behaviours. For instance, unwanted DNA self-hybridization and cross hybridization^30^ would decrease the local order of DNA layer and undermine the effect of orientational attraction. Moreover, surface heterogeneity can widen the melting curve, enhancing the negative cooperativity^12^. On the other side, aggregation on the surface was reported during the preparation of both dsDNA and ssDNA monolayers, giving dense and spare domains^42^,^43^,^44^. This implies the existence of favourable interactions between same kind of DNA, which would promote positive cooperativity and phase separation. Depletion effect may also have the same effect^17^. We also note that molecular crowding conditions can change the water activity, thus affects the solvation of DNA and thermodynamic of DNA hybridization^48^,^49^. In addition, the rearrangement of water's hydrogen-bonds network and change of local dielectric properties in the crowding situation is also expected to modify the DNA hybridization cooperativity^1^.

In fact, a more sensitive system to investigate the DNA cooperative hybridization is the liquid DNA brush system, in which tethered DNA molecules have mobility on the surface. Experimental researches in this direction have just gained its momentum^45^,^46^,^47^. Since the adding of the mobility usually promotes phase separation as well as weakening cross-hybridization and surface heterogeneity, hybridization cooperativity can be more easily perceived. The detecting sensitivity can also be improved by using DNA tetrahedral nanostructures as demonstrated by Lin et al.^50^.

## Conclusion

In this work, we systematically investigate the molecular crowding and its effect on DNA surface hybridization. We find molecular crowding can lead to two types of cooperativity due to the competition between various interactions. Generally speaking, DNA molecules feel isotropic excluded volume and orientational interaction, electrostatic repulsion, hydration repulsion and osmotic pressure from ions in the layer. It is found that the crowding-strengthened repulsions cause the negative cooperativity, while the entropy-favouring orientational attraction is the driving force for positive cooperativity. Under certain conditions, this positive cooperativity can induce a first order phase separation on surface. We discussed various factors that affect DNA surface hybridization, including DNA molecular density, ion strength, surface curvature and DNA chain length. Our discovery is not only important for practical applications, but also of great significance to understand complex crowding-induced biological phenomena in cell.

## Methods

In the present work, we focus on the thermodynamics of DNA surface hybridization based on the assumption that two ssDNA hybridize into one perfect dsDNA(two-state model). In our model, unhybridized ssDNA molecules are tethered on planar or spherical surface, shown in Figure 1. Hybridization happens when a tethered ssDNA captures its complementary ssDNA in solution and turns into a rigid dsDNA. The coil state ssDNA, which has the persistence length of 2 *nm*^51^, is described by the worm-like chain model(see Supplementary Section 5). While helix state dsDNA is modeled as rigid rod, which can rotate freely on its anchored point. Due to counterion condensation effect, dsDNA is assumed to take 0.75 *e*^−^ charge per nucleotide pair, while ssDNA takes 0.5 *e*^−^ per nucleotide, according to recent experimental results^52^. Cations (*Na*^+^) and anions (*Cl*^−^) are explicitly included and are assumed to have a hydrated radius of 0.35 nm, while solvents only enter the theory implicitly through the dielectric constant *ε* = 78^53^.

We use a molecular theory^36^,^54^ that explicitly considers the size, rigidity, conformation, charge, and inter-molecular interactions between all molecular species in the system. The theory is formulated by writing down the free energy of the system. In general terms, it can be expressed as
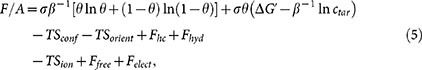
where *A* is the surface area of the system; *σ* is the molecular density of DNA; Δ*G*′ is the surface hybridization free energy; *c_tar_* is the target ssDNA concentration. The first term describes the associating entropy of DNA between two states (hybridized and unhybridized); *S_conf_* represents the configurational entropy of flexible ssDNA, while *S_orient_* represents the orientational entropy of dsDNA helix. *F_hc_* is the helix-coil excluded volume interaction^41^,^55^ between DNA molecules, which consists of isotropic and orientation-dependent parts. *F_hyd_* is the short-range hydration repulsion between parallel dsDNA. *S_ion_* is the entropy of ions, and *F_free_* is its free volume modification due to the existence of crowded DNA. The last *F_elect_* represents the electrostatic energy of the system. Each of these terms is a function of distributions of the different molecular species, charge, the probabilities of the dsDNA and ssDNA conformations. We minimize *F* with respect to these functions to determine the equilibrium structure of the layer. In Supplementary Information, we present a detailed description of the molecular model, the free energy expression, the minimization procedure, etc.

## Supplementary Material

Supplementary Information Supplementary Information
