# Supplementary material for: Crowding-induced Cooperativity in DNA Surface Hybridization
Source: Sci Rep. 2015 Apr 15;5:9217. doi: 10.1038/srep09217 (PMC5381746; doi:10.1038/srep09217)
Supplement: Supplementary Information [file srep09217-s1.pdf]

Supplementary Information

for "Crowding-induced Cooperativity in DNA Surface Hybridization"

Qun-li Lei[†], Chun-lai Ren[†,*], Xiao-hang Su[†] and Yu-qiang Ma[†,‡,*]

[†] *National Laboratory of Solid State Microstructures and Department of Physics, Collaborative Innovation Center of Advanced Microstructures, Nanjing University, Nanjing 210093, China*

‡ *Center for Soft Condensed Matter Physics and Interdisciplinary Research, Soochow University, Suzhou 215006, China*

∗ *E-mail: chunlair@nju.edu.cn. (C. R.); myqiang@nju.edu.cn. (Y. M.)*

1. Model and Theory

For simplicity, we first consider the low DNA surface coverage case, where interactions among DNA molecules can be neglected. The free energy per molecule can be written as

$$\frac{\beta F}{A\sigma} = \theta\ln\theta + (1-\theta)\ln(1-\theta) + \theta(\beta\Delta G' - \beta\mu_{tar}), \tag{1}$$

where F is the free energy of system, A is the surface area, σ is DNA surface coverage and θ is the hybridization fraction of DNA molecules. μ_{tar} is the chemical potential of complementary ssDNA in the solution, which satisfies $\mu_{tar} = \beta^{-1}\ln c_{tar}$. Here c_{tar} is the ssDNA concentration in the solution. The first two terms in Eq. (1) are the associating entropy between dsDNA and ssDNA states. The energy change arising from single DNA hybridization is characterized by surface hybridization free energy $\Delta G'$. It satisfies the relationship of $\Delta G' = \Delta G_0 + \Delta U_0$, where ΔG_0 is the standard hybridization free energy in DNA solution and ΔU_0 is the difference between potentials of mean force(PMF) of a isolate dsDNA and ssDNA on surface. In the ideal situation ΔU_0 mainly comes from the conformational deformation due to existence of surface. Eq. (1) is only suitable for the ultra-spare DNA layer, where DNA molecules hybridize or dehybridize independently of each other. The result is the classical Langmuir isotherm[1,2]

$$\frac{\theta}{1-\theta} = c_{tar} \cdot e^{-\beta\Delta G'} \ . \tag{2}$$

When DNA molecules are grafted with a high surface coverage, they form the structure of brush. Flexible ssDNA chains in this situation incline to adopt a more stretched configuration, while rod-like dsDNA molecules tend to be perpendicular to the surface. The entropic terms aroused by their conformational change are denoted as conformational entropy S_{conf} and orientational entropy S_{orient} for ssDNA and dsDNA respectively, which are given by:

$$S_{conf} = -k_B \sum_{\alpha_{ss}} P_{ss}(\alpha_{ss}) \ln P_{ss}(\alpha_{ss}) \tag{3}$$

$$S_{orient} = -k_B \sum_{\alpha_{ds}} P_{ds}(\alpha_{ds}) \ln P_{ds}(\alpha_{ds}), \tag{4}$$

where $P(\alpha)$ is the probability that a tethered molecule in configuration of α. Subscripts ds and ss represent dsDNA and ssDNA respectively.

For the mixture of dsDNA and ssDNA, excluded volume interactions between helices and coils can be described by Flory's theory of rod-coil mixtures[3]. Since we consider the inhomogeneous surface system, this part of the free energy can be written as[4]

$$\begin{aligned}\frac{v_{ss}\cdot F_{hc}(r)}{k_BT} &= [1-\phi_{ss}(r)-\phi_{ds}(r)]\ln[1-\phi_{ss}(r)-\phi_{ds}(r)] \\ &\quad -[1-\lambda(r)\phi_{ds}(r)]\ln[1-\lambda(r)\phi_{ds}(r)] \\ &\quad +[1-\lambda(r)]\phi_{ds}(r)+\phi_{ss}(r), \end{aligned} \tag{5}$$

where $\phi_{ds}(r)$ and $\phi_{ss}(r)$ are local volume fractions of dsDNA and ssDNA. λ is Flory's orientational order parameter, which is defined by $\lambda=(1-<\sin\theta_\alpha>)$, where $<>$ denotes the average and θ_α is the angle between a specific helix and domain axis[3]. The first term in Eq. (5) represents isotropic excluded volume effect since it only depends on volume fraction. The rest λ-contained terms describe orientation-dependent excluded volume effect. The physical meaning of Eq. (5) can be seen clearly if we do the expansion and keep the second order(second virial) terms under the condition of low concentration, which can be expressed as:

$$\frac{F_{hc}(r)}{k_BT} \simeq v_{ss}c_{ss}^2+2v_{ds}c_{ss}c_{ds}+(1-\lambda^2)Lv_{ds}c_{ds}^2, \tag{6}$$

where c_{ss} and c_{ds} are concentrations of ssDNA segment and dsDNA molecule, and v_{ss} and v_{ds} are the excluded volume parameters respectively. $L=v_{ds}/v_{ss}$ can be viewed as the aspect ratio of the helix. Therefore, F_{hc} is a decreasing function of $\lambda(r)\phi_{ds}(r)$, which implies that more ordered dsDNA are energetically preferred.

Flory's model assumed that steric repulsions between two parallel rods are negligible. However, for two close parallel dsDNA, hydration repulsions begin to play an important role. The repulsive energy per base pair follows[5]:

$$V_{dd}=\frac{al_{bp}e^{-d/r_H}}{\sqrt{2d/\pi r_H}}, \tag{7}$$

where d is the distance between the axes of two parallel dsDNA. $r_H=0.3\ nm$ is the characteristic decay length for hydration interaction, and $l_{bp}=0.34\ nm$ is the length of a base pair. Interaction parameter a is chosen as $1.0\times10^{-7}\ J/m^5$.

As for the description of electrostatic energy, we adopt a modified Poisson-Boltzmann equation proposed by Borukhov and Andelman[6], where the excluded volumes of ions are considered. Thus, the electrostatic contribution to the total free energy is

$$\frac{F_{eletr}}{k_BT} = \int d\mathbf{r}\beta[-\frac{1}{2}\epsilon[\nabla\psi(r)]^2+\rho_{char}(r)\psi(r)-\rho_+(r)\mu_+-\rho_-(r)\mu_-] \tag{8}$$

with local charge density

$$\rho_{char}(r) = -z_{ss}\rho_{ss}(r) - z_{ds}\rho_{ds}(r) + z_+\rho_+(r) - z_-\rho_-(r), \tag{9}$$

where z_i and $\rho_i(r)$ denote the charge valence and local number density of the i component respectively. If the charge of DNA is simply taken as its bare charge, then we will get $z_{ss} = 1$ (per nucleotide) for ssDNA and $z_{ds} = 2$ (per nucleotide pair) for dsDNA . However, due to the effect of counterion condensation, z_{ss} and z_{ds} can be much smaller[7–10]. Recent experimental evidences[10] have shown that the effective charge ratio between dsDNA and ssDNA molecules is about $z_{ds}/z_{ss} = 0.75/0.5$. We choose this ratio for our calculations.

In Borukhov and Andelman's original description[6], the entropic contribution from ions is

$$-\frac{S_{ions}}{k_B} = \frac{1}{v_\pm}\int d\mathbf{r}[\phi_+(r)\ln\phi_+(r) + \phi_-(r)\ln\phi_-(r) + \phi_{sol}(r)\ln\phi_{sol}(r)], \tag{10}$$

where $\phi_\pm(r) = \rho_\pm(r)v_\pm$ are the local volume fraction of cations or anions. $\phi_{sol}(r)$ denotes the local volume fraction of solvent. The first two terms in the integral represent the ideal translational entropy of ions, while the third term accounts for the inter-ions excluded volume interactions. It should be mentioned that, in our system, the space that ions can move freely is reduced due to the existence of DNA molecules. The free energy penalty arising from this crowding effect can be written as[11]:

$$\frac{F_{free}}{k_BT} = -\frac{1}{v_\pm}\int d\mathbf{r}[\phi_+(r) + \phi_-(r) + \phi_{sol}(r)]\ln\phi_{free}(r), \tag{11}$$

where $\phi_{free}(r) = \phi_+(r) + \phi_-(r) + \phi_{sol}(r)$ denotes the free volume fraction, that is, excluding the volume fraction occupied by DNA.

Importantly, Eq. (5), (10), (11) together constitute the total mixing entropy of the system, where the translational entropy of ions and the excluded volume interactions between DNA-DNA, ion-ion and DNA-ion are all included.

Excluded volume parameters for a nucleotide in ssDNA chain is $v_{ss} = 0.5\ nm^3$. Volume of a nucleotide pair in dsDNA is assumed two times of v_{ss}, i.e. $v_{ds} = 1.0\ nm^3$. For cation and anion, $v_\pm = v_{ss}/3$, which corresponds to a hydration shield with radius of 0.35 nm[12]. For simplicity, free ssDNA molecules in the solution are omitted in our calculation, since the concentration is usually around $10^{-5}M$ and their influence on the surface can be ignored[13]. Furthermore, we take the mean field approximation and assume that inhomogeneous distributions of different components only exist in the direction perpendicular to the surface r.

In the Section 2, the free energy of the whole system will be minimized and equilibrium distributions of each components are obtained. Here we just show the final DNA surface hybridization equation, namely,

$$\frac{\theta}{1-\theta} = \frac{q_{ds}(\theta)}{q_{ss}(\theta)} \cdot \frac{q_{ss0}}{q_{ds0}} \cdot c_{tar} \cdot e^{-\beta\Delta G_0}, \tag{12}$$

where $q_{ss}(\theta)$ and $q_{ds}(\theta)$ are single chain partition functions of ssDNA and dsDNA molecules in the DNA layer . q_{ss0} and q_{ds0} are ones for ssDNA and dsDNA molecules in the bulk solution. Eq. (12) can be transformed into a more general equation of DNA hybridization isotherm

$$\frac{\theta}{1-\theta} = c_{tar} \cdot e^{-\beta[\Delta G_0 + \Delta G_{ex}(\theta)]}. \tag{13}$$

where $\Delta G_{ex} = U_{ds} - U_{ss}$ can be taken as an excess hybridization free energy. $\beta U_{ds} = -\ln[q_{ds}(\theta)/q_{ds0}]$ and $\beta U_{ss} = -\ln[q_{ss}(\theta)/q_{ss0}]$ is the potentials of mean force(PMF) of dsDNA and ssDNA staying in DNA layer(moved from bulk solution).

The expression of FWHM of DNA melting curve in solution takes a simple form[14]

$$W_0 = \frac{4k_B T_m^2}{\Delta H_0}. \tag{14}$$

For our DNA surface hybridization system, according to Eq. 13, the width can be obtained from

$$W = \left. \frac{(4 + \beta \frac{\partial}{\partial\theta}\Delta G_{ex})}{\Delta H_0 + \Delta U_m} k_B T_m^2 \right|_{\theta=0.5}, \tag{15}$$

where ΔH_0 is the enthalpic part of ΔG_0 and $\Delta U_m = -\frac{\partial}{\partial\beta} \ln \frac{q_{ds}}{q_{ss}}$. From the expression of q_{ds} and q_{ss} (see minimization procedure), it can be found that ΔU_m has two sources: electrostatic potential and hydration repulsion. These two energy contributions are estimated to be order of 1 k_BT per nucleotide pair under the conditions we concern. On the other hand, ΔH_0 is about 8 $kcal\ mol^{-1}$ (15 k_BT) per nucleotide pair[15]. Thus, generally $\Delta H_0 \gg \Delta U_m$ and Eq. (15) can be simplified as

$$W \simeq D \cdot W_0 \tag{16}$$

with a normalized FWHM denoted by

$$D = 1 + \left. \frac{\beta}{4} \frac{\partial \Delta G_{ex}(\theta)}{\partial\theta} \right|_{\theta=0.5}. \tag{17}$$

Note that the exchange chemical potential[16] of single DNA molecule from coil to helix state $\Delta\mu = \mu_{ds} - \mu_{ss}$ is

$$\begin{aligned} \beta\Delta\mu &= \frac{\beta\partial W}{A\sigma\partial\theta} \\ &= \ln\frac{\theta}{1-\theta} - \ln c_{tar} + \beta\Delta G_0 + \beta\Delta G_{ex}(\theta). \end{aligned} \quad (18)$$

Thus, D can be written into a more general form

$$D = \beta\,\frac{1}{4}\frac{\partial\Delta\mu}{\partial\theta}\bigg|_{\theta=0.5}. \quad (19)$$

2. Equilibrium state

The system studied in this work is a semi-open system, thus the accurate characteristic function to determine thermodynamic behavior is semi-grand potential[17], although in the main paper we simply call it free energy. In last section, we explicitly analyze different contributions to the semi-grand potential. There are two constraints for the system that are not mentioned:

$$\phi_{ds}(r) + \phi_{ss}(r) + \phi_{free}(r) = 1 \quad (20)$$

$$\phi_{+}(r) + \phi_{-}(r) + \phi_{sol}(r) - \phi_{free}(r) = 0. \quad (21)$$

These two constraints together describe the incompressibility of liquid which are fulfilled by introducing Lagrange multipliers $\Pi(r)$ and $\pi(r)$. The full version of semi-grand potential

W with these Lagrange multipliers is

$$\begin{aligned}\frac{\beta W}{A} &= \sigma\theta\left[\sum_{\alpha_{ds}} P_{ds}(\alpha_{ds})\ln P_{ds}(\alpha_{ds}) + \ln q_{ds0}\right] \\ &+\sigma(1-\theta)\left[\sum_{\alpha_{ss}} P_{ss}(\alpha_{ss})\ln P_{ss}(\alpha_{ss}) + \ln q_{ss0}\right] \\ &+\sigma\left[\theta\ln\theta + (1-\theta)\ln(1-\theta)\right] + \sigma\theta\left(\beta\Delta G_0 - \beta\mu_{tar} + \beta V_{hyd}\right) \\ &+\frac{1}{v_\pm}\sum_{i=+,-,sol}\int \phi_i(r)\left[\ln\phi_i(r) - \ln\phi_{free}(r) - \beta\mu_i\right]dr \\ &+\int\left[\rho_q\beta\psi(r) - \frac{1}{2}\varepsilon\beta(\nabla\psi(r))^2\right]dr \\ &+\frac{1}{v_{ss}}\int F_{hc}[\phi_{free}(r),\phi_{ds}(r),\phi_{ss}(r)]dr \\ &+\int \beta\Pi(r)\left[\phi_{ss}(r)+\phi_{ds}(r)+\phi_{free}(r)-1\right]dr \\ &+\int \beta\pi(r)\left[\phi_+(r)+\phi_-(r)+\phi_{sol}(r)-\phi_{free}(r)\right]dr, \end{aligned} \tag{22}$$

where F_{hc} is given by Eq. (5). V_{hyd} is hydration repulsion between dsDNA molecules, which is written in a continual way,

$$V_{hyd} = \int \frac{3\phi_{ds}(r)}{\theta\sigma v_{ds}} V_{dd}(r)dr \tag{23}$$

with $V_{dd}(r) = \frac{a\ l_{bp}e^{-d(r)/r_H}}{\sqrt{2d/\pi r_H}}$ and $d(r) = \left(\frac{2v_{ds}}{\sqrt{3}\phi_{ds}(r)l_{bp}}\right)^{1/2}$, corresponding to hexagonal lattice packing for every discrete layers.

The minimization of the semi-grand potential with respect to different variables gives the thermal equilibrium distributions of each component and DNA configuration probability. For example, the molecular densities of cations and anions can be obtained as

$$\rho_+(r) = \rho_{+,bulk}\cdot\phi_{free}(r)\cdot e^{-\beta\pi(r)v_\pm - \beta z_+\psi(r)} \tag{24}$$

$$\rho_-(r) = \rho_{-,bulk}\cdot\phi_{free}(r)\cdot e^{-\beta\pi(r)v_\pm + \beta z_-\psi(r)}, \tag{25}$$

where $\rho_{\pm,bulk}$ are bulk concentration of cation or anion. Expressions for free volume fraction and volume fractions of the solvent background are

$$\phi_{free}(r) = e^{-\beta\Pi(r)v_\pm} \tag{26}$$

$$\phi_{sol}(r) = \phi_{sol,bulk}\cdot\phi_{free}(r)\cdot e^{-\beta\pi(r)v_\pm}. \tag{27}$$

The physical meanings of $\Pi(r)$ and $\pi(r)$ are discussed in Section 4.

In our calculation, dsDNA is assumed as rigid rod which can rotate freely upon its anchored point. A rotating dsDNA possesses 72×72 configurations characterized by different polar and azimuth angles. For ssDNA, 10^5 configurations are generated by Monte Carlo method based on worm-like chain model[18]. Probability distribution functions for ssDNA and dsDNA configurations can be obtained as

$$P_{ss}(\alpha_{ss}) = \frac{1}{q_{ss}} e^{-\beta\int n_{ss}(r,\alpha_{ss})U_{ss}^{ex}(r)dr} \tag{28}$$

$$P_{ds}(\alpha_{ds}) = \frac{1}{q_{ds}} e^{-\beta\int n_{ds}(r,\alpha_{ds})U_{ds}^{ex}(r)dr} \tag{29}$$

with

$$U_{ss}^{ex}(r) = \Pi(r)v_{ss} + \pi(r)v_{ss} + z_{ss}\psi(r) \tag{30}$$

$$\begin{aligned} U_{ds}^{ex}(r) &= \Pi(r)v_{ds} + \pi(r)v_{ds} + z_{ds}\psi(r) \\ &\quad -2\beta^{-1}(\sin\theta_{\alpha_{ds}} - 1)\ln[1-\lambda\phi_{ds}(r)] \\ &\quad +\frac{V_{dd}(r)}{2}[5 + d(r)/r_H], \end{aligned} \tag{31}$$

where $n(r,\alpha)$ represents segments distribution of a specific conformation α. U_{ss}^{ex} and U_{ds}^{ex} can be viewed as the external potential felt by a DNA nucleotide(pair) at position r.

Variation with respect to $\psi(r)$ gives rise to a generalized Poisson-Boltzmann equation:

$$\epsilon\nabla^2\psi(r) = -\langle\rho_q(r)\rangle. \tag{32}$$

In our model, we use the following boundary conditions:

$$\lim_{r\to\infty}\psi(r) = 0 \tag{33}$$

$$\left.\frac{\partial\psi(r)}{\partial r}\right|_{r=0} = 0, \tag{34}$$

The second boundary condition corresponds to uncharged surface.

At last, when minimizing W against hybridization fraction θ, we get the final DNA hybridization equation

$$\frac{\theta}{1-\theta} = \frac{q_{ds}(\theta)}{q_{ss}(\theta)}\cdot\frac{q_{ss0}}{q_{ds0}}\cdot c_{tar}\cdot e^{-\beta\Delta G_0}. \tag{35}$$

The minimized free energy thus is

$$\begin{aligned}\frac{\beta W_{min}}{A} &= -\sigma \ln Q - \beta \int \Pi(r)dr - \beta \int \pi(r)dr \\ &\quad -\frac{1}{v_{ss}} \int \{\phi_{ss}(r) + [\lambda+1]\phi_{ds}(r) - \ln[1-\lambda\phi_{ds}(r)]\}dr \\ &\quad +\beta \int \{\frac{\phi_{ds}(r)V_{dd}(r)}{2v_{ss}}[1+d(r)/r_H] + \frac{1}{2}\varepsilon[\nabla\psi(r)]^2\}dr \\ &\quad +const. \end{aligned} \tag{36}$$

Q can be viewed as the overall partition function of the DNA molecule, given by

$$Q = q_{ss} + q_{ds} \cdot c_{tar} \cdot e^{-\beta\Delta G_0 - \ln q_{ds0} + \ln q_{ss0}}. \tag{37}$$

Surface tension of DNA layer is calculated by[19]

$$\Pi_s = \beta\sigma \frac{\partial W_{min}}{A\partial\sigma} - \frac{\beta W_{min}}{A}. \tag{38}$$

Details of the free energy minimization, discretization and numerical methods can be found in previous publications[17,20]. It should be mentioned that our theory differs from the previous one in two points. One is that the solvent implicit model used here, and the other is that we use a free volume approximation to account for the size asymmetry effect between large DNA segment volume and small ions[11].

3. Potential of mean force

We take the case of dsDNA molecule as a example. The expression of U_{ds}^{ex} (Eq.(31)) indicates that a dsDNA molecule in DNA layer need to bear the isotropic excluded volume and orientational interactions between DNA molecules, ionic osmotic pressure, electrostatic interaction and hydration repulsion, namely

$$U_{ds}^{ex}(r) = U_{iso}(r) + U_{osm}(r) + U_{ele}(r) + U_{ori}(r) + U_{hyd}(r) \tag{39}$$

with

$$U_{iso}(r) = \Pi(r)v_{ds} \tag{40}$$

$$U_{osm}(r) = \pi(r)v_{ds} \tag{41}$$

$$U_{ele}(r) = z_{ds}\psi(r) \tag{42}$$

$$U_{ori}(r) = -2\beta^{-1}(\sin\theta_{\alpha_{ds}} - 1)\ln[1-\lambda\phi_{ds}(r)] \tag{43}$$

$$U_{hyd}(r) = \frac{V_{dd}(r)}{2}[5+d(r)/r_H] \tag{44}$$

The average potential felt by a dsDNA molecule, or the potential of mean force(PMF) from external mean field, can be calculated according to the probability distribution, namely

$$\begin{aligned} U_{ds}^{ex} &= \sum_{\alpha_{ds}}\left[\int n_{ds}(r,\alpha_{ds})U_{ds}^{ex}(r)dr\right]P_{ds}(\alpha_{ds}) \\ &= -\beta^{-1}\ln q_{ds} + TS_{orient} \end{aligned} \tag{45}$$

PMF arising from conformational deformation is

$$U_{ds}^{def} = -T(S_{orient} - S_{orient,bulk}) \tag{46}$$

With $S_{orient,bulk} = -k_B \ln q_{ds0}$ the undisturbed orientational entropy of dsDNA in bulk solution. Therefore, the the total PMF to move a dsDNA from bulk solution(free state) to DNA layer(grafted state) is

$$U_{ds} = U_{ds}^{ex} + U_{ds}^{def} = -\beta^{-1}\ln q_{ds}/q_{ds0} \tag{47}$$

4. The Physical Meaning of π and Π

π and Π are two Lagrange multipliers introduced by our theory to fulfil the incompressibility constraint of liquid. Both of them have the dimension of pressure, which can be expressed as

$$\beta\Pi = \frac{1}{v_{ss}}\ln\phi_{free} = \frac{1}{v_{ss}}\ln(1-\phi_{ss}-\phi_{ds}) \tag{48}$$

$$\beta\pi = \frac{1}{v_{\pm}}\ln\frac{\phi_{sol}}{\phi_{free}} = \frac{1}{v_{\pm}}\ln(1-\phi'_{+}-\phi'_{-}). \tag{49}$$

For simplicity, we omit the position dependent symbol (r) here. From Eq. (48), we can get $\beta\Pi\ v_{ss} = \ln(1-\phi_{ss}-\phi_{ds})$, which represents the energy required to create a cavity of volume v_{ss} in the environment of crowded DNA chains (without ions)[21]. Similarly, we can write $\beta\pi v_{\pm} = \ln(1-\phi'_{+}-\phi'_{-})$ with $\phi'_{\pm} = \phi_{\pm}/\phi_{free}$, which implies the energy needed to create a cavity of volume $v_{\pm}$ in the surrounding of ions. Here the denominator ϕ_{free} is a free volume correction for the actual volume available to ions due to the excluded volume of DNA. Therefore, in our theory , Π solely comes from excluded volume interactions between large DNA segments, while π arises from small mobile ions. The latter is the osmotic pressure induced by ions. It should be mentioned that the additivity of π and Π in our theory comes from the free volume approximation and is only valid for system with high asymmetry in molecule size. When $v_{ss} \approx v_{\pm}$, ions are randomly mixed with chain segments. Discrimination of ionic contribution to the osmotic pressure is meaningless.

5. Worm-like Model of DNA

In the model, we describe ssDNA with worm-like model. The bending energy associated with a particular conformation of a discrete chain is[22]

$$E_{bend} = -\varepsilon \sum_{n=1}^{N-1} \vec{t}_n \cdot \vec{t}_{n+1} = -\varepsilon \sum_{n=1}^{N-1} \cos\theta_n \tag{50}$$

with $\varepsilon b = k_B T l_p$, where b is the discrete length, l_p is the persistence length. θ_n is the angle between $\vec{t}_n$ and $\vec{t}_{n+1}$. The bending probability is[23]

$$P(\vec{t}_n, \vec{t}_{n+1}) = q^{-1} \sin\theta_n \exp\left[\frac{\varepsilon\cos\theta_n}{k_B T}\right] \tag{51}$$

Monte Carlo method is used to product the chain configuration of order 10^5. To test the validity of this method, we calculate chain's end to end distribution $<R^2>$, and find it converge well to analytical result of classical work-like model[18]

$$<R^2> = 2l_p R_{max} - 2l_p^2 \left(1-\exp(-\frac{R_{max}}{l_p})\right) \tag{52}$$

where R_{max} is the contour length of chain molecule.

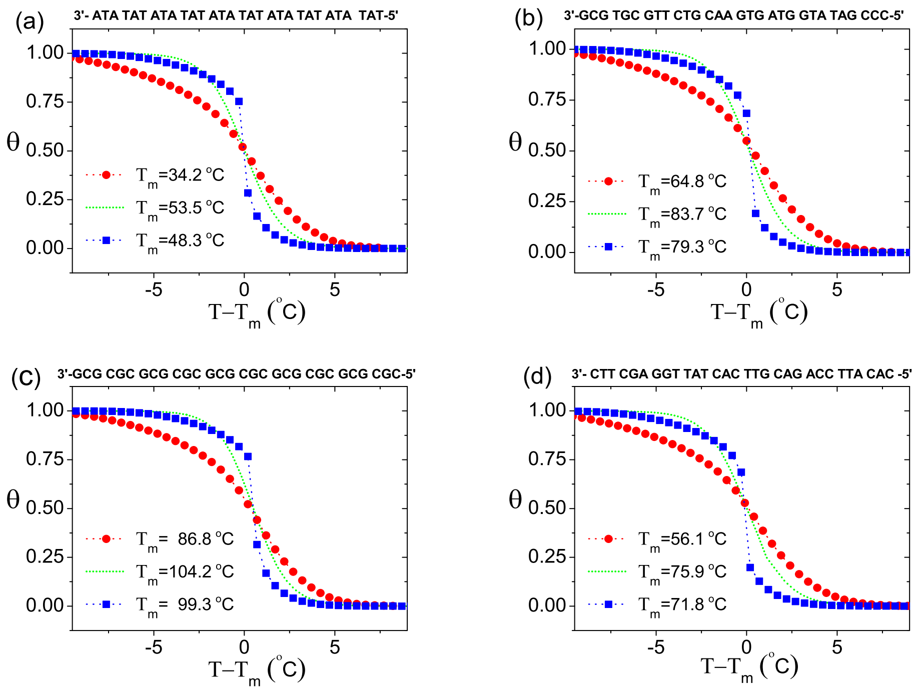

Fig. S 1: **Melting curves for different DNA sequence.** The red and blue lines indicate the negative, and positive cooperativity respectively, as Figure 2 (in the manuscript) shows. The probe sequences used to calculate the melting curves are:

(a). 5'- ATA TAT ATA TAT ATA TAT ATA TAT ATA TAT -3' (pure A-T sequence)

$\Delta H_0 = -204.2\ kcal\ mol^{-1}$, $\Delta S_0 = -597.0\ cal\ mol^{-1} \cdot K^{-1}$, $c_{tar} = 100\ nM$

(b). 5'-GCG TGC GTT CTG CAA GTG ATG GTA TAG CCC-3' (G-C rich sequence)

$\Delta H_0 = -246.0\ kcal\ mol^{-1}$, $\Delta S_0 = -660.4\ cal\ mol^{-1} \cdot K^{-1}$, $c_{tar} = 100\ nM$

(c). 5'-GCG CGC GCG CGC GCG CGC GCG CGC GCG CGC-3' (pure G-C sequence)

$\Delta H_0 = -296.0\ kcal\ mol^{-1}$, $\Delta S_0 = -755.3\ cal\ mol^{-1} \cdot K^{-1}$, $c_{tar} = 100\ nM$

(d). 5'- CTT CGA GGT TAT CAC TTG CAG ACC TTA CAC -3' (random sequence)

$\Delta H_0 = -223.3\ kcal\ mol^{-1}$, $\Delta S_0 = -611.6\ cal\ mol^{-1} \cdot K^{-1}$, $c_{tar} = 100\ nM$.

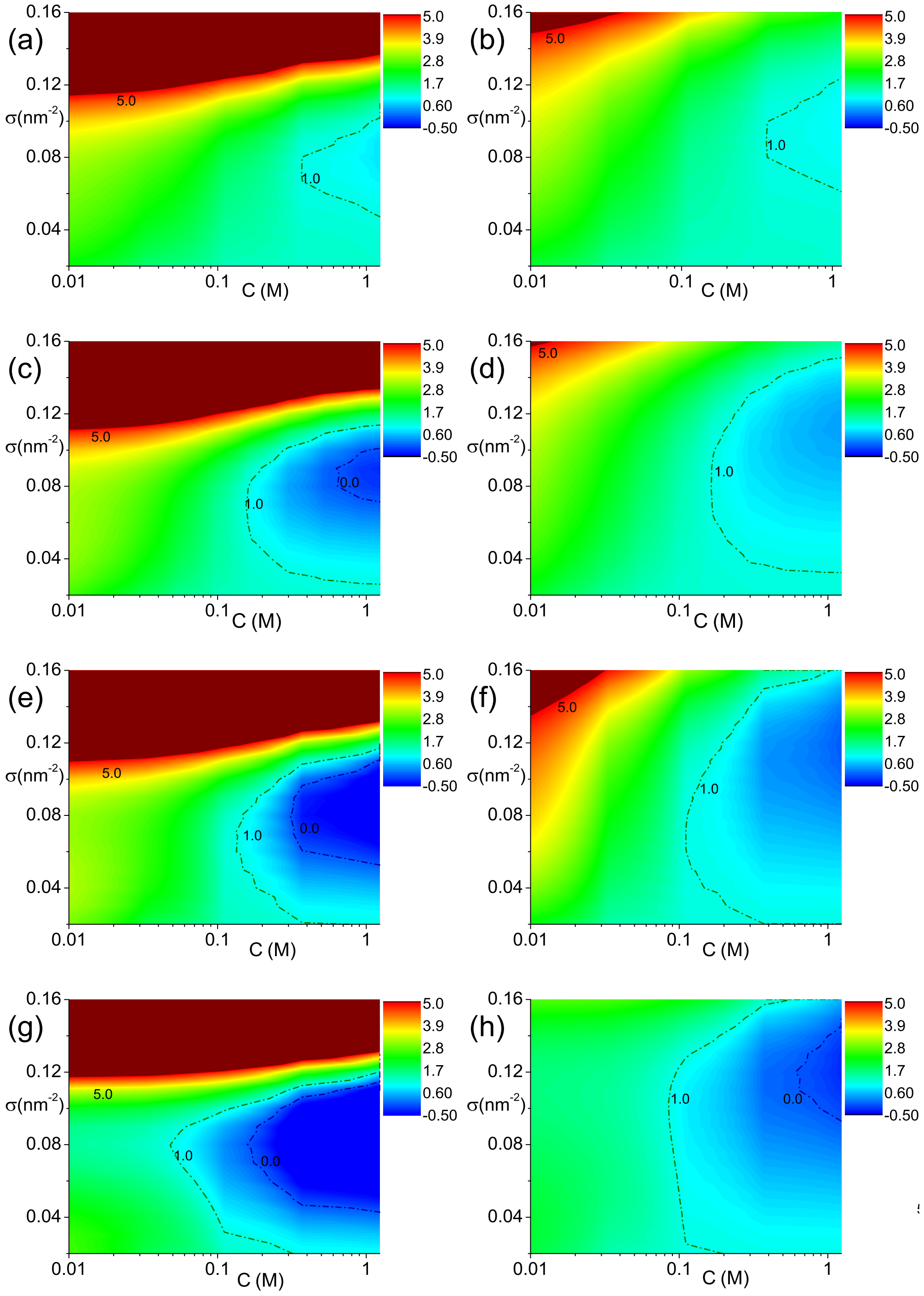

Fig. S 2: **Cooperativity phase diagrams for different DNA lengths.** Left column represents the case of planar surface and right column is the condition of spherical surface with the radius of 10 nm. From top to bottom, DNA length N=20, 30, 40 and 50.

[1] Langmuir, I. The Adsorption of Gases On Plane Surfaces of Glass, Mica and Platinum. *J. Am. Chem. Soc.* **40**, 1361–1403 (1918).

[2] Vainrub, A. & Pettitt, B. M. Coulomb blockage of hybridization in two-dimensional DNA arrays. *Phy. Rev. E* **66**, 041905 (2002).

[3] Flory, P. J. Statistical Thermodynamics of Mixtures of Rodlike Particles .5. Mixtures With Random Coils. *Macromolecules* **11**, 1138–1141 (1978).

[4] Kastantin, M. & Tirrell, M. Helix Formation in the Polymer Brush. *Macromolecules* **44**, 4977–4987 (2011).

[5] Strey, H. H., Parsegian, V. A. & Podgornik, R. Equation of state for DNA liquid crystals: Fluctuation enhanced electrostatic double layer repulsion. *Phys. Rev. Lett.* **78**, 895–898 (1997).

[6] Borukhov, I., Andelman, D. & Orland, H. Steric effects in electrolytes: A modified Poisson-Boltzmann equation. *Phys. Rev. Lett.* **79**, 435–438 (1997).

[7] Spasic, A. & Mohanty, U. Counterion Condensation In Nucleic Acid. *Adv. Chem. Phys.* **139**, 139–176 (2008).

[8] Raspaud, E., da Conceicao, M. & Livolant, F. Do free DNA counterions control the osmotic pressure? *Phys. Rev. Lett.* **84**, 2533–2536 (2000).

[9] Rant, U. *et al.* Excessive counterion condensation on immobilized ssDNA in solutions of high ionic strength. *Biophys. J.* **85**, 3858–3864 (2003).

[10] Vuletic, T. *et al.* Manning free counterion fraction for a rodlike polyion: Aqueous solutions of short DNA fragments in presence of very low added salt. *Phy. Rev. E* **83**, 041803 (2011).

[11] Popovic, M. & Siber, A. Lattice-gas Poisson-Boltzmann approach for sterically asymmetric electrolytes. *Phy. Rev. E* **88**, 022302 (2013).

[12] Ren, C. L., Tian, W. D., Szleifer, I. & Ma, Y. Q. Specific Salt Effects on Poly(ethylene oxide) Electrolyte Solutions. *Macromolecules* **44**, 1719–1727 (2011).

[13] Vainrub, A. & Pettitt, B. M. Sensitive quantitative nucleic acid detection using oligonucleotide microarrays. *J. Am. Chem. Soc.* **125**, 7798–7799 (2003).

[14] Everaers, R., Kumar, S. & Simm, C. Unified description of poly- and oligonucleotide DNA melting: Nearest-neighbor, Poland-Sheraga, and lattice models. *Phy. Rev. E* **75**, 041918 (2007).

[15] SantaLucia, J. & Hicks, D. The thermodynamics of DNA structural motifs. *Annu. Rev. Biophys.*

Biomol. Struct. **33**, 415–440 (2004).

[16] Halperin, A., Buhot, A. & Zhulina, E. B. Sensitivity, specificity, and the hybridization isotherms of DNA chips. *Biophys. J.* **86**, 718–730 (2004).

[17] Nap, R., Gong, P. & Szleifer, I. Weak polyelectrolytes tethered to surfaces: Effect of geometry, acid-base equilibrium and electrical permittivity. *J. Poly. Sci. B* **44**, 2638–2662 (2006).

[18] Rubinstein, M. & Colby, R. *Polymer Physics* (Oxford University Press, USA, 2003).

[19] Carignano, M. A. & Szleifer, I. Pressure Isotherms, Phase-transition, Instability, and Structure of Tethered Polymers In Good, Theta, and Poor Solvents. *J. Chem. Phys.* **100**, 3210–3223 (1994).

[20] Ren, C. L. & Ma, Y. Q. Loading oligonucleotides on a nanoparticle regulated by a grafted polyethylenimine layer. *Soft Matter* **7**, 10841–10849 (2011).

[21] Kumar, S. K., Szleifer, I. & Panagiotopoulos, A. Z. Determination of the Chemical-potentials of Polymeric Systems From Monte-carlo Simulations. *Phys. Rev. Lett.* **66**, 2935–2938 (1991).

[22] Mazur, A. K. Wormlike chain theory and bending of short DNA. *Phys. Rev. Lett.* **98**, 218102 (2007).

[23] Rappaport, S. M. & Rabin, Y. Model of DNA bending by cooperative binding of proteins. *Phys. Rev. Lett.* **101**, 038101 (2008).
